# Annotations

**Published:** 1895-07-06

**Authors:** 


### Annotations.

### The Notification of Measles.

Notwithstanding all our advances in sanitation, the loss of life from measles remains untouched. When the Acts were passed which rendered compulsory the notification of infectious diseases, measles was exempted from their operation, for it seemed impossible by notification to catch the cases early enough for isolation to be of any use, while it seemed hopeless to think of providing sufficient hospital accommodation to isolate a disease which was so widespread. It has been said from the beginning, and it still is said, that notification and isolation must go hand in hand if the benefits of either are to be experienced. But as a fact, in dealing with zymotic disease, notification does good independently of hospitals, and hospital treatment does good independently of isolation. As a rule it is the children of the poor who die, and, curiously enough, it is not so much of measles that they die as of complications. In the crowded—and often insanitary—dwellings of the poor, the catarrh which is the constant accompaniment of measles takes on a septic form, and the patient dies of broncho-pneumonia or exhausting diarrhœa. These complications are certainly due to dirt, closeness, and generally deficient sanitation, and it is by no means certain that if notification were followed by a compulsory cleaning it might not be almost as useful as if it led to compulsory removal. We may yet live to see the municipal charwoman. What happened at Hull during the cholera time, when on each notification Dr. Mason's "sanitary column," with its mops and pails, took possession of the house and cleaned it down, would be of great service in dealing with other diseases, and would often be far cheaper than prolonged treatment in hospital. Of course, there are cases where surroundings are so bad, and accommodation so deficient, that removal to hospital is necessary. But if we got rid of the "fetish" of isolation, kept the child in hospital only so long as treatment was required, and meanwhile cleaned the home, we should probably get far more out of our notification than we should do by retaining the patient an indefinite time for purely isolation purposes, and then sending it back to its dirt. Something, however, should be done. Dr. Waldo has recently sent in a report to the vestry of St. George, Southwark, drawing attention to the great loss of life from measles, 3,291 persons having died from that complaint in London during 1894, or 7·5 per 1,000 persons living. He advocates notification; so do we. But we consider that the benefits to be derived from it are largely thrown away if it is made a mere stepping-stone to the hospital.

### Huxley's Aim in Life.

To know, after a great man is dead, what he really aimed at in life, is one of the most efficient of all aids to the understanding of the man himself. It will probably be the opinion of posterity that few men of the nineteenth century were better worth understanding than Huxley. In his autobiographical introduction to his collected essays, recently published, the dead scientist tells the world what he wanted to do for it and with himself. "If I may speak of the objects I have had more or less definitely in view since I began the ascent of my hillock," he says, "they are, briefly, these: To promote the increase of natural knowledge, and to forward the application of scientific methods of investigation to all the problems of life to the best of my ability, in the conviction, which has grown with my growth and strengthened with my strength, that there is no alleviation for the sufferings of mankind except veracity of thought and of action, and the resolute facing of the world as it is." What Huxley saw most clearly of all things was the tremendous struggle which everywhere accompanies human life, or, to use his own phrase, "the sufferings of mankind." What he desired to do was to mitigate the struggle, or, again in his own phrase, to "alleviate the sufferings." This is not the picture of him which most of us have been accustomed to look at, but it is the true picture. And how did he hope to alleviate the sufferings of mankind? Many other men have had his aim, but their own methods of reaching it. What was Huxley's method? "Veracity of thought and action"; a "resolute facing of the world as it is." Other methods there may be, and are. Huxley's will bear comparison with most of them. He was a great man, with a great method: a hero of science and of modern times. The world is the richer by his life, and the poorer by his death. All that is honest and true and clearsighted in the nation and the world will mourn for him.

### Medical Defence and Protection.

The Medical Defence Union and the London and Counties Medical Protection Society, after more than twelve months of almost unintermittent negotiation, appear to be still but at the beginning of their labours There would seem to be a want of statesmanship somewhere. On the first blush the amalgamation of the two societies appears to be so eminently desirable that the observer is inclined to be a little impatient. Why should there be two medical societies having one and the same object? There ought not to be two if one would do the work as well or better than the two. But in practice it is found that the council of one of the societies, which meets once a month, has such a plethora of work at every meeting that it is almost impossible ever to get any subject discussed with thoroughness. But if this be the case when two societies divide the work between them, how will the work be overtaken at all if amalgamation should be carried through, and all that is now done by two should be thrown upon a single council? This is an aspect of the case which is overlooked by those who see only that amalgamation would result in an increase of numbers, and consequently of financial strength, to one society. We sincerely desire amalgamation between the two societies if it can be carried out without impairing working efficiency. The only way in which fusion can really serve the profession is by the establishment of a united and strong central society in London, to which all cases should be sent that cannot be dealt with locally, and the creation of competent branches at all important centres of population, which shall undertake all work of a purely local character. Medical defence and protection has a most useful future before it if those who are now at the head of affairs in London will only show a moderate amount of capacity for practical statesmanship.

